# Destigmatisation of Mental Health Conditions: The Use of Social Media in the Holistic Education of Medical Students

**DOI:** 10.1192/bjo.2025.10268

**Published:** 2025-06-20

**Authors:** Jenat Dhliwayo

**Affiliations:** Northern Ireland Medical & Dental Training Agency, Belfast, United Kingdom

## Abstract

**Aims:** Medical students’ stigma towards mental health conditions is a well-documented phenomenon in the scientific literature – recommendations to combat this include utilising a holistic approach when teaching about mental health conditions. The holistic approach can be done in a multitude of ways to deliver mental health topics in a multifaceted way. With the increase in social media consumption, there has been a significant rise in consumer exposure to mental health-based content. This content ranges from aetiology and diagnostic information to first-person accounts of living with mental health conditions, highlighting the wide range of discussion points provided by social media platforms.

The aim of this research is to determine whether there is a place for the use of social media in the holistic approach in the destigmatisation of mental health conditions in medical students.

**Methods:** A positivism-influenced approach was used to conduct anonymised two-part survey, using mostly a quantitative approach (60%). Participants were asked to fill out ‘yes, no, maybe, don’t know’ questions about whether they think social media could be used to teach them in medical school about mental health conditions. The remaining 40% qualitative part of the survey, allowed participants to detail their experiences of mental health on social media and express their thoughts on if and how they thought social media could be used to educate medical students. 10 participants took part in this anonymised survey. All participants were based in medical schools in Northern Ireland, in their fourth or final year of medical school.

**Results:** All participants reported encountering mental health-based content on social media. 90% of those participants believed that social media contributed positively to their overall opinion on mental health conditions. 80% of participants believed that social media should play a role in educating medical students about mental health conditions. Suggestions on how social media can be used in the education of medical students include showing first-person accounts of what it is like to live with mental health conditions and also showing accounts from family and friends who deal/dealt with mentally ill loved ones.

**Conclusion:** There is a space for the use of social media in the holistic approach to achieve the destigmatisation of mental health conditions in medical students. Social media can be used to drive empathy-based reflective practices in students via the utilisation of first-person experiences from the mentally unwell people themselves or their loved ones.

